# Development of a Mouse Reporter Strain for the Purinergic P2X_2_ Receptor

**DOI:** 10.1523/ENEURO.0203-20.2020

**Published:** 2020-08-05

**Authors:** Seol-Hee Kim, Parmvir K. Bahia, Mayur Patil, Sydney Sutton, Isobel Sowells, Stephen H. Hadley, Marian Kollarik, Thomas E. Taylor-Clark

**Affiliations:** Molecular Pharmacology & Physiology, Morsani College of Medicine, University of South Florida, Tampa, FL 33612

**Keywords:** ATP, P2X2, reporter mouse, sensory neurons, tracing

## Abstract

The ATP-sensitive P2X_2_ ionotropic receptor plays a critical role in a number of signal processes including taste and hearing, carotid body detection of hypoxia, the exercise pressor reflex and sensory transduction of mechanical stimuli in the airways and bladder. Elucidation of the role of P2X_2_ has been hindered by the lack of selective tools. In particular, detection of P2X_2_ using established pharmacological and biochemical techniques yields dramatically different expression patterns, particularly in the peripheral and central nervous systems. Here, we have developed a knock-in P2X_2_-cre mouse, which we crossed with a cre-sensitive tdTomato reporter mouse to determine P2X_2_ expression. P2X_2_ was found in more than 80% of nodose vagal afferent neurons, but not in jugular vagal afferent neurons. Reporter expression correlated in vagal neurons with sensitivity to α,β methylene ATP (αβmATP). P2X_2_ was expressed in 75% of petrosal afferents, but only 12% and 4% of dorsal root ganglia (DRG) and trigeminal afferents, respectively. P2X_2_ expression was limited to very few cell types systemically. Together with the central terminals of P2X_2_-expressing afferents, reporter expression in the CNS was mainly found in brainstem neurons projecting mossy fibers to the cerebellum, with little expression in the hippocampus or cortex. The structure of peripheral terminals of P2X_2_-expressing afferents was demonstrated in the tongue (taste buds), carotid body, trachea and esophagus. P2X_2_ was observed in hair cells and support cells in the cochlear, but not in spiral afferent neurons. This mouse strain provides a novel approach to the identification and manipulation of P2X_2_-expressing cell types.

## Significance Statement

Inhibitor and knock-out studies have demonstrated the critical role of P2X_2_ in multiple sensory signaling pathways. Nevertheless, P2X_2_ expression patterns are controversial, as biochemical studies suggest widespread expression, whereas functional studies suggest restricted expression. Functional characterization is further complicated by heteromeric P2X_2/3_ channels that have hybrid pharmacology and biophysical properties. We have developed a P2X_2_-cre mouse to determine the expression pattern of P2X_2_. In the periphery, P2X_2_ expression is found in almost all nodose sensory afferents but is limited to only minor subsets of trigeminal and DRG afferents. Centrally, P2X_2_ is mostly expressed in neurons projecting mossy fibers to the cerebellum. Thus, we provide novel evidence for the specific expression of P2X_2_, which is more limited than previously thought.

## Introduction

P2X_2_ is one of seven members of the P2X purinergic receptor family (Brake et al., 1994; Dunn et al., 2001; Khakh et al., 2001). P2X_2_ is a cation-permeable plasma membrane ion channel activated by extracellular ATP, which can form either functional homomeric channels or functional heteromeric channels with P2X_3_ (Brake et al., 1994; Chen et al., 1995; Lewis et al., 1995). Based on pharmacological and genetic knock-out studies, P2X_2_ plays important roles in a number of peripheral organs, including taste cell signaling to gustatory sensory afferents (Finger et al., 2005; Huang et al., 2011), hypoxic signaling in carotid bodies (Rong et al., 2003), protection from noise-induced ototoxicity (Yan et al., 2013), the exercise pressor reflex (McCord et al., 2010), and mechanical transduction in sensory afferents innervating the airways (Weigand et al., 2012) and bladder (Cockayne et al., 2005). Nevertheless, the precise role of P2X_2_ in peripheral afferents and in other systems is hindered by the lack of selective tools.

P2X_2_ and P2X_3_ homomeric channels can be discriminated by the desensitization of their ATP-evoked currents (limited desensitization/persistent currents for P2X_2_ channels, rapid desensitization for P2X_3_) and their sensitivity to α,β methylene ATP (αβmATP; P2X_3_ is activated by this ATP analog, whereas P2X_2_ is not; Dunn et al., 2001; Khakh et al., 2001). Nevertheless, co-expression of P2X_2_ and P2X_3_ causes the formation of heteromeric P2X_2/3_ channels that evoke mixed/persistent currents in response to both ATP and αβmATP (Lewis et al., 1995). Furthermore, the kinetics of P2X channel desensitization are modulated by numerous factors (Bianchi et al., 1999; Sokolova et al., 2006), thus decreasing its effectiveness as a diagnostic for discriminating P2X_2_, P2X_3_, and P2X_2/3_ channels. Biochemical detection of P2X_2_ reveals similar inconsistencies: immunohistochemistry and *in situ* hybridization has revealed robust and widespread expression of P2X_2_ in peripheral and central neurons and smooth muscle (Brake et al., 1994; Kidd et al., 1995; Collo et al., 1996; Vulchanova et al., 1996; Kanjhan et al., 1999; Petruska et al., 2000b; Yao et al., 2000, 2003; Gourine et al., 2003; Spehr et al., 2004; Ambalavanar et al., 2005; Cockayne et al., 2005; Simonetti et al., 2006; Staikopoulos et al., 2007; Song et al., 2012), although functional studies of ATP- and αβmATP-evoked currents suggest a much more limited expression pattern. As such there is considerable uncertainty regarding the expression of P2X_2_, despite its established role in cellular signaling, particularly in the peripheral nervous system (Rong et al., 2003; Cockayne et al., 2005; Finger et al., 2005; McCord et al., 2010; Huang et al., 2011; Weigand et al., 2012).

Here, we used genetic targeting of the endogenous P2X_2_ locus to generate a knock-in reporter mouse that expresses Cre recombinase in P2X_2_-expressing cells. After crossing this strain with a cre-sensitive tdTomato reporter mouse strain, we visualized P2X_2_ expression in sensory ganglia, the CNS and in peripheral tissues. We found robust reporter expression in nodose vagal neurons but not in jugular vagal neurons, and reporter expression was limited to few neurons in the trigeminal ganglia and dorsal root ganglia (DRG). We confirmed the expression of P2X_2_ in tdTomato-expressing vagal neurons by assessing Ca^2+^ influx in response to the P2X_2/3_ agonist αβmATP using fura-2 AM. Reporter expression was used to visualize P2X_2_-expressing terminals in the tongue, carotid body, trachea and esophagus as well as in the nucleus tractus solitarius (nTS) in the dorsal medulla (the location of central terminations of nodose sensory afferents). Elsewhere in the CNS, reporter expression was largely limited to medullary and pontine neurons protecting mossy fibers to the cerebellum, although reporter expression was also noted in a small number of cerebellar Purkinje neurons, cerebral cortical neurons and caudoputamen neurons. Lastly, we observed expression in hair cells and support cells in the organ of Corti in the cochlea. Thus, this reporter mouse demonstrates the specific expression of the purinergic receptor P2X_2_ and provides a novel tool to study the structure and function of these particular cells.

## Materials and Methods

### Knock-in mouse model development

**Figure 1.**
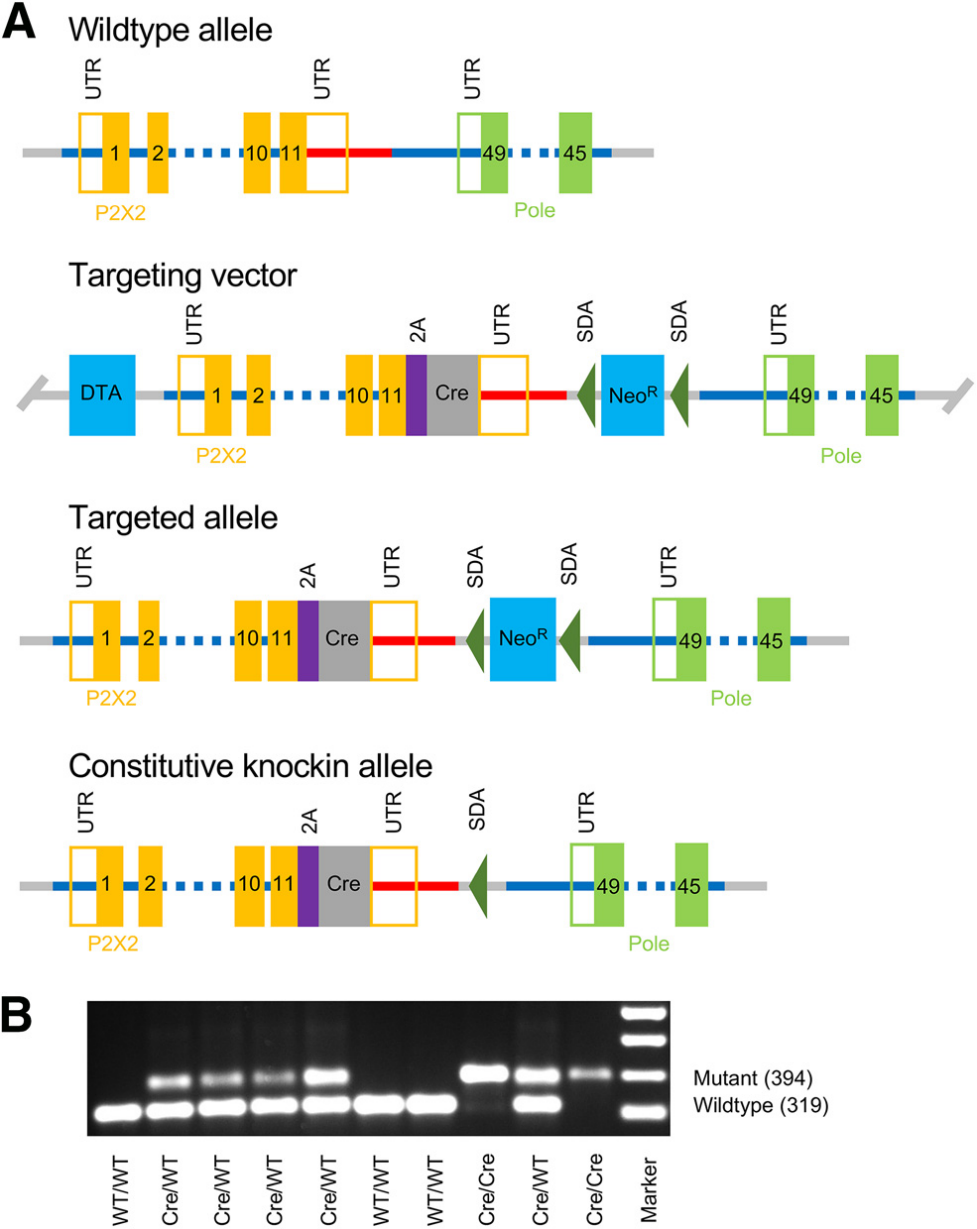
Development of the knock-in P2X_2_^Cre^ mouse. ***A***, Targeting strategy for the replacement of the P2X_2_ TGA stop codon with a 2A-Cre cassette (2A self-cleaving peptide in purple, Cre in dark gray). Homology arms (blue and red lines) were generated for the P2X_2_ gene [exons, 5′ and 3′ untranslated regions (UTRs) in orange] and the latter portion of the neighboring Pole gene (exons, and 3′ UTR in green). Homology arms of targeting vector include a neomycin resistance gene (Neo^R^) flanked by SDA sites for positive selection. A Diphtheria toxin A fragment gene (DTA) was placed in a non-homologous region of targeting vector as a negative selection for non-homologous recombination. ***B***, PCR of P2X_2_ gene in offspring from a pairing of heterozygous P2X_2_^Cre^ mice. As expected, offspring have a mendelian distribution of mutant (i.e., P2X_2_^Cre^, at 394 bp) and wild-type (at 319 bp) alleles.

The gene for the murine P2X_2_ receptor (*P2rx2* gene, NCBI Reference Sequence: NM_153400.4) is located on chromosome 5. Eleven exons have been identified, with the ATG start codon in exon 1 and TGA stop codon in exon 11. In order to develop a knock-in mouse that expresses Cre recombinase dependent on P2X_2_ expression, the P2X_2_ TGA stop codon was replaced with a 2A-Cre cassette (Fig. 1). The targeting vector homology arms were generated by high fidelity Taq PCR using BAC clone RP23-333M22 and RP23-354O18 from the C57BL/6J library as template. The targeting vector was assembled with recombination sites and selection markers: neomycin resistance gene (Neo^R^) flanked by self-deletion anchor (SDA) sites for positive selection and diphtheria toxin A fragment gene (DTA) for negative selection. Correct targeting vector synthesis was confirmed by appropriate digestion by restriction enzymes. The linearized vector was subsequently delivered to C57BL/6 ES cells via electroporation, followed by drug selection, PCR screening, and Southern blotting confirmation. After gaining 94 neomycin-resistant clones, 18 potentially targeted clones were confirmed, five of which were expanded for Southern blotting. After confirming correctly targeted ES clones via Southern blotting, clones were selected for blastocyst microinjection, followed by founder production. Founders were confirmed as germline-transmitted via crossbreeding with wild type. All aspects of knock-in mouse development were performed by Cyagen US Inc (California). Founders were mated to produce heterozygous and homozygous mice (*P2rx2^tm1.1(cre)Ttc^*, MGI:2665170) in expected Mendelian proportions. These mice express P2X_2_-2A-Cre from the endogenous P2X_2_ gene. Upon translation, the 2A peptide self-cleaves (Furler et al., 2001) to release P2X_2_ and Cre as separate peptides. *P2rx2^tm1.1(cre)Ttc^* mice develop normally and were observed to have no apparent phenotype. Homozygous *P2rx2^tm1.1(cre)Ttc^* were crossed with the ROSA26-loxP-STOP-loxP-tdTomato mice (*B6.Cg-Gt(ROSA)26Sortm9(CAG-tdTomato)Hze/J*, #007909, The Jackson Laboratory) to produce *P2X_2_^Cre/+^/ROSA26-tdTomato^fl/+^* (P2X_2_-tdTomato mice), with cell-specific expression of tdTomato via Cre recombination. Specific alleles were confirmed by genotyping per developers’ instructions. Both male and female mice (six to eight weeks old) were used for experiments. Offspring were weaned at 21 postnatal days and up to four littermates were housed per cage under normal condition (20°C, a 12/12 h light/dark cycle). Mice were provided with standard rodent chow and water *ad libitum*. All procedures were in accordance with the animal protocol approved by the Institutional Animal Care and Use Committee.

### Tissue collection and immunofluorescence

Mice were euthanized by CO_2_ inhalation and transcardially perfused with ice-cold PBS followed by perfusion fixation with ice-cold 3.7% formaldehyde (FA). Vagal ganglia, petrosal ganglia, trigeminal ganglia, thoracic DRG (T_1_-T_5_), and carotid body were dissected out and postfixed for 1 h in 3.7% FA at 4°C. Medulla, spinal cord, and tongue were collected and postfixed for 4 h in 3.7% FA at 4°C. Brains were collected and postfixed overnight in 3.7% FA at 4°C. For the cochlea, the temporal bones were collected and postfixed in 4% PFA overnight at 4°C. The temporal bones were washed three times in PBS for 10 min and transferred to 10% EDTA (dissolved in H_2_O, pH 7.2) solution for overnight decalcification at 4°C. Tissue firmness was checked by pressing tissue with forceps to ensure adequate decalcification.

All tissues were washed in PBS to remove residual FA and transferred to 20% sucrose solution for cryoprotection. Tissue were mounted in optimal cutting temperature (OCT) compound and snap frozen in dry-ice before cryosectioning: sensory ganglia (20 μm), carotid bodies (20 μm), tongue (40 μm), cochlea (40 μm), spinal cord (20 μm), medulla (40 μm for coronal, 80 μm for sagittal), and brain (80 μm). All slices were collected onto Superfroast plus slides. Slides were then air-dried at room temperature in the dark overnight. For immunofluorescence, sectioned tissue was permeabilized with 0.3% Triton X-100 in PBS (PBSTx) for 15 min followed by blocking with 1% bovine serum albumin (BSA)/10% donkey serum (DS)/0.3% PBSTx. Sectioned tissue was incubated with primary antibodies diluted in blocking buffer overnight at 4°C. Sensory ganglia slices were stained for immunoreactivity to TRPV1 (goat, 1:150, sc-12 498, Santa Cruz), the neurotrophin receptors tyrosine receptor kinase A (TRKA; rabbit, 1:300, 06-574, Millipore) or pgp9.5 (rabbit, 1:300, AB5925, Millipore). Carotid body slices were immunostained against tyrosine hydroxylase (TH; rabbit, 1:300, AB112, Abcam). Sequentially sectioned medulla slices were stained against TRPV1 (guinea pig, 1:150, GP14100, Neuromics). After washing with 0.2% Tween 20 in PBS (PBST) three times for 10 min, tissue was incubated with appropriate secondary antibodies [chicken anti-goat 647 (1:300, A212345, Invitrogen), donkey anti-rabbit 488 (1:300, A21206, Invitrogen), and donkey anti-guinea pig 647 (1:300, AP193SA6, Millipore)] in 1% BSA/5% DS in 0.2% PBST for 1 h. Tissue was washed with 0.2% PBST three times for 10 min and rinsed briefly with H_2_O. In some cases, sectioned tissue was counterstained with either green or blue fluorescent Nissl staining (1:600, NeuroTrace 500/525 or 435/455, Fluorescent Nissl Stain, Invitrogen). Slides were air dried and mounted with DPX mounting medium (Sigma) or mounted with VECTASHIELD Antifade Medium with DAPI (Vector laboratories).

### Wholemount immunostaining of trachea and esophagus

The mouse trachea was split lengthwise by making a single cut through the midline. The esophagus was cut around the stomach and then split lengthwise. The mucosal and muscle layers of the esophagus were separated carefully by small sharp dissection scissors. We stained the trachea by first permeabilizing with 1% Tween 20 (Sigma-Aldrich) in filtered 1× PBS for 6 h at room temperature, then washed three times for 20 min in filtered 1× PBS using rotator. Permeabilized tracheal tissues were incubated with rabbit anti-RFP primary antibody (1:200, 600-401-379, Rockland antibodies) in PBS with 1% BSA for 48 h at 4°C (repositioned five to six times during incubation), washed in filtered 1× PBS ten times using rotator at 4°C, incubated with secondary antibody goat-anti-rabbit Alexa Fluor 568 (Invitrogen) with dilution 1:100 in filtered 1× PBS for 5 h at room temperature. Trachea were then washed in filtered 1× PBS (three times, rotator, 4°C), incubated in anti-fade glycerol (pH 8.6, 10× Tris-buffered saline mixed with glycerol) for 24 h at room temperature and stored in anti-fade glycerol at 4°C. The esophageal tissues went through the same treatment as the trachea, but the main difference was that we amplified the tdTomato signal by using a streptavidin-biotin protocol. In short, after the primary antibody step, the tissues were washed in 1× PBS and then incubated with goat biotin‐XX conjugate anti-rabbit IgG (H + L) secondary antibody (1:100, B2770, Invitrogen) in 1% PBS/BSA overnight at room temperature (repositioned one to two times during incubation), washed in filtered 1× PBS 10 times using rotator at 4°C, and incubated with streptavidin conjugated with fluorescent dye Alexa Fluor 568 (Invitrogen) with dilution 1:100 in filtered 1× PBS for 5 h at room temperature. All stained tissues were then positioned muscular or mucosal side up on a glass slide and covered with coverslip 24 × 50 mm. In the first stained preparation, we found numerous randomly distributed oval solid artifacts. Optimization of staining procedure revealed that these artifacts attributable to the use of 1% goat blocking serum. In control experiments, omitting goat serum eliminated these artifacts, while including goat serum reproduced them. Therefore, in all subsequent staining, goat serum was omitted.

### Visualization of reporter expression in sectioned and wholemount tissue

Images were taken with either Olympus FV1200 laser scanning confocal microscope or Andor Dragonfly spinning disk confocal microscope using Fusion software, and projection images were processed with Imaris software. In all cases the identification of anatomic structures and subnuclei were based on the mouse brain map (Paxinos and Franklin, 2012). *Z* stack images (10× and 20×) of the mouse esophagus and trachea (wholemount) were taken using Andor Dragonfly spinning disk microscope. Nerve terminals in the epithelial and subepithelial layers of the trachea were traced using Neurolucida 360 software (MBF Biosciences), as previously described (Dickstein et al., 2016): the 20× images were spaced at 0.6 μm. First, automatic nerve terminal detection (Rayburst crawl) was used, seeds were validated, then manual tracing was used to complete the tracing.

### Vagal ganglia dissociation

Male 6- to 12-week-old P2X_2_-tdTomato mice were euthanized by CO_2_ asphyxiation followed by exsanguination. As previously described (Stanford and Taylor-Clark, 2018), vagal ganglia were isolated in Ca^2+^-free, Mg^2+^-free HBSS, then incubated in HBSS containing collagenase (2 mg/ml) and dispase (2 mg/ml), then mechanically dissociated with fire-polished pipettes. Individual neurons were washed with L-15 media supplemented with 10% fetal bovine serum, 100 U/ml penicillin, and 100 μg/ml streptomycin, then plated onto poly-D-lysine and laminin-coated coverslips. Neurons were incubated at 37°C in antibiotic-free L-15 media supplemented with 10% fetal bovine serum and used within 24 h.

### Live neuron Ca^2+^ imaging and analysis

Neurons were incubated with 4 μm fura-2 AM for 30–60 min at 37°C. Coverslips were loaded into a chamber on an inverted microscope and perfused with heated (33–34°C) HEPES buffer (154 mm NaCl, 1.2 mm KCl, 1.2 mm MgCl_2_, 2.5 mm CaCl_2_, and 5.6 mm D-glucose). Slides equilibrated for 10 min before the start of the experiment and an image was taken to visualize tdTomato fluorescence (535-nm excitation, 610-nm emission). Changes in [Ca^2+^]_i_ was monitored using sequential excitation at 340 and 380 nm (510-nm emission) with images taken every 6 s using a CoolSnap HQ2 camera (Photometrics) and evaluated ratiometrically using the 340/380 ratio. All drugs were diluted in HEPES buffer. αβmATP (10 μm) was used to determine the functional expression of P2X_2/3_ channels (Taylor-Clark et al., 2015). The EC_50_ for αβmATP-evoked activation of P2X_2/3_ channels is ∼3–9 μm (Khakh et al., 2001; Liu et al., 2001). Capsaicin (1 μm) was used to determine the functional expression of the nociceptive ion channel TRPV1 (Taylor-Clark et al., 2015). Neurons were further characterized by response to KCl (75 mm) before ionomycin (5 μM), which evoked a maximal Ca^2+^ response. Image analysis was performed by using Nikon Elements (Nikon) by drawing individual regions of interest (ROIs) that around the intracellular region for each cell and tracked over time. ROIs with an unstable, high, or noisy baseline were eliminated from analysis. Neurons which failed to exhibit an increase in [Ca^2+^]_i_ to either αβmATP, capsaicin or KCl challenges (>30% the ionomycin maximal response) were eliminated. Relative changes in [Ca^2+^]_i_ were determined ratiometrically using fura-2 fluorescence: 340/380 ratio (R). This negates the impact of cell to cell variations in fura-2 AM loading. Data are presented as changes in the 340/380 ratio (Δ*R* = R_1_ – R_0_), where R0 is the average 340/380 ratio before mATP treatment. As before (Stanford and Taylor-Clark, 2018; Stanford et al., 2019), an individual neuron was considered to be sensitive to a given agent if R_agent_ > (R_bl_ + 2*SD_bl_) + 0.075, where R_agent_ is the average 340/380 ratio during treatment, R_bl_ is the average 340/380 ratio before treatment, and SD_bl_ is the SD of R_bl_. Neurons were grouped by tdTomato expression and sensitivity to αβmATP and capsaicin. Data and statistical analyses were performed using Microsoft Excel and GraphPad Prism 7. Mean Ca^2+^ responses were compared using Student’s *t* test; *p* = 0.05 was taken as the threshold for significance.

## Results

To investigate the expression of P2X_2_, we generated a knock-in P2X_2_^Cre^ mouse (Fig. 1), which was crossed with the cre-sensitive ROSA26-loxP-STOP-loxP-tdTomato reporter mouse. The resultant P2X_2_-tdTomato mice express the red fluorescent tdTomato in P2X_2_-expressing cells.

**Figure 2.**
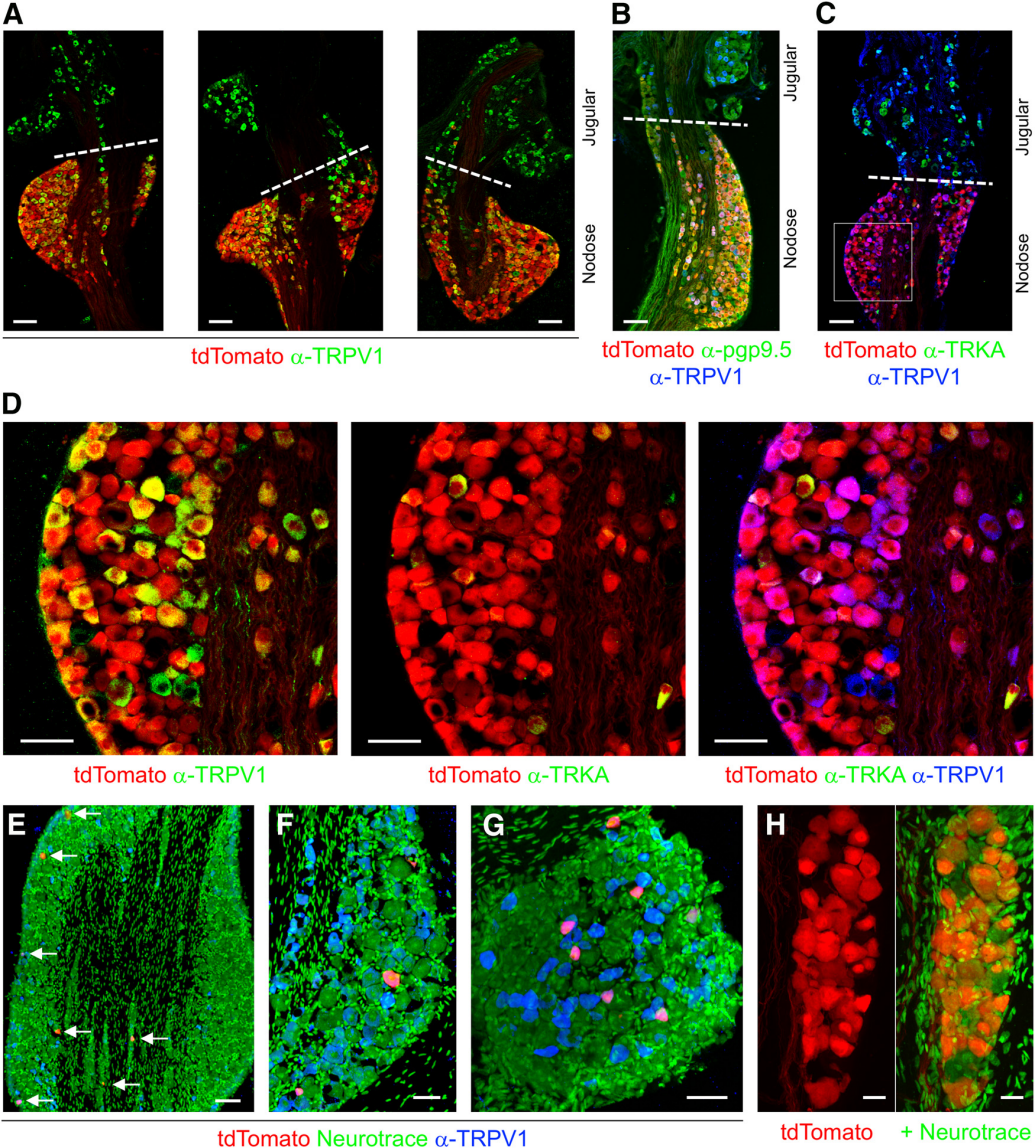
tdTomato expression in P2X_2_-tdTomato mice is restricted to subsets of sensory afferents. ***A–C***, tdTomato (red) expression in vagal ganglia, with the demarcation of the nodose and jugular regions delineated by dotted line. ***A***, Counterstained for TRPV1 (green) expression. ***B***, Counterstained for TRPV1 (blue) and pgp9.5 (green) expression. ***C***, Counterstained for TRPV1 (blue) and TRKA (green) expression. ***D***, tdTomato (red) expression in nodose neurons identified in ***C***. Left, Counterstained for TRPV1 (green) expression. Middle, Counterstained for TRKA (green) expression. Right, Counterstained for TRPV1 (blue) and TRKA (green) expression. ***E–G***, tdTomato (red) expression counterstained for TRPV1 expression (blue) and with NeuroTrace (green). ***E***, Maxillary/ophthalmic region of the trigeminal ganglia. tdTomato+ neurons identified by white arrows. ***F***, Mandibular region of the trigeminal ganglia. ***G***, DRG. ***H***, Petrosal ganglia, counterstained with NeuroTrace (green). Scale bars: 100 μm (***A–C***, ***E***), 50 μm (***D***, ***F***, ***G***), and 20 μm (***H***). Data are derived from five vagal ganglia, five trigeminal ganglia, four DRG, and four petrosal ganglia.

**Figure 3.**
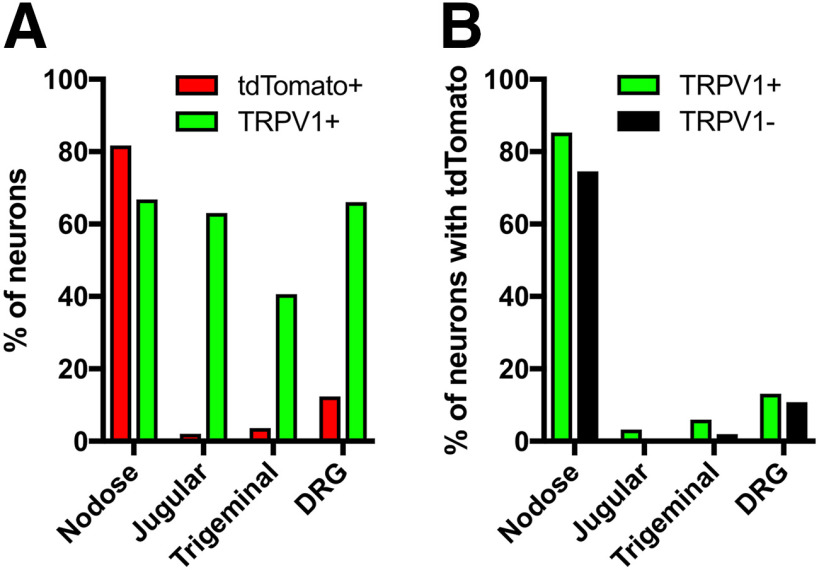
Quantification of tdTomato and TRPV1 expression in vagal, trigeminal, and DRG afferents. ***A***, The % of neurotrace+ neurons that express tdTomato and TRPV1. ***B***, The % of TRPV1+ neurons and TRPV1– neurons that also express tdTomato. Data are derived from two vagal ganglia, five trigeminal ganglia, and three thoracic DRG.

Cryostat sections of the vagal ganglia from four P2X_2_-tdTomato mice showed robust and selective expression of tdTomato in nodose vagal sensory neurons, whereas there was little tdTomato expression in jugular vagal sensory neurons (1045 vs 16 tdTomato+ neurons, respectively; Fig. 2*A*). Vagal ganglia from two of these P2X_2_-tdTomato mice were assessed for NeuroTrace (Nissl) staining (data not shown) to calculate the percentage of neurons that expressed tdTomato. More than 80% of nodose neurons were tdTomato+, compared with just 2% of jugular neurons (Fig. 3*A*). This is consistent with data from vagal ganglia from P2X_2_-tdTomato stained for the neuronal marker pgp9.5, which again showed that few nodose neurons failed to express tdTomato (Fig. 2*B*). tdTomato expression in nodose neurons was noted in >75% of both TRPV1+ and TRPV1– populations (Figs. 2*A-D*, 3*B*). Very few tdTomato+ neurons also expressed TRKA, which was widely expressed in jugular neurons (Fig. 2*C*,*D*). We then investigated tdTomato expression in the trigeminal and thoracic DRG of P2X_2_-tdTomato mice. In sections counterstained with NeuroTrace, we found only 3.6% of trigeminal neurons were tdTomato+, and these were equally distributed between the maxillary/ophthalmic and mandibular regions (Figs. 2*E*,*F*, 3*A*). The rare tdTomato+ trigeminal neurons were mostly TRPV1+ (Figs. 2*E*,*F*, 3*B*). Few DRG neurons (12.3%) expressed tdTomato (Figs. 2*G*, 3*A*), although these were equally split between TRPV1+ and TRPV1– populations (Figs. 2*G*, 3*B*). Lastly, we found extensive tdTomato expression within the petrosal ganglia, with 75% of petrosal neurons from four ganglia expressing the marker (Fig. 2*H*).

**Figure 4.**
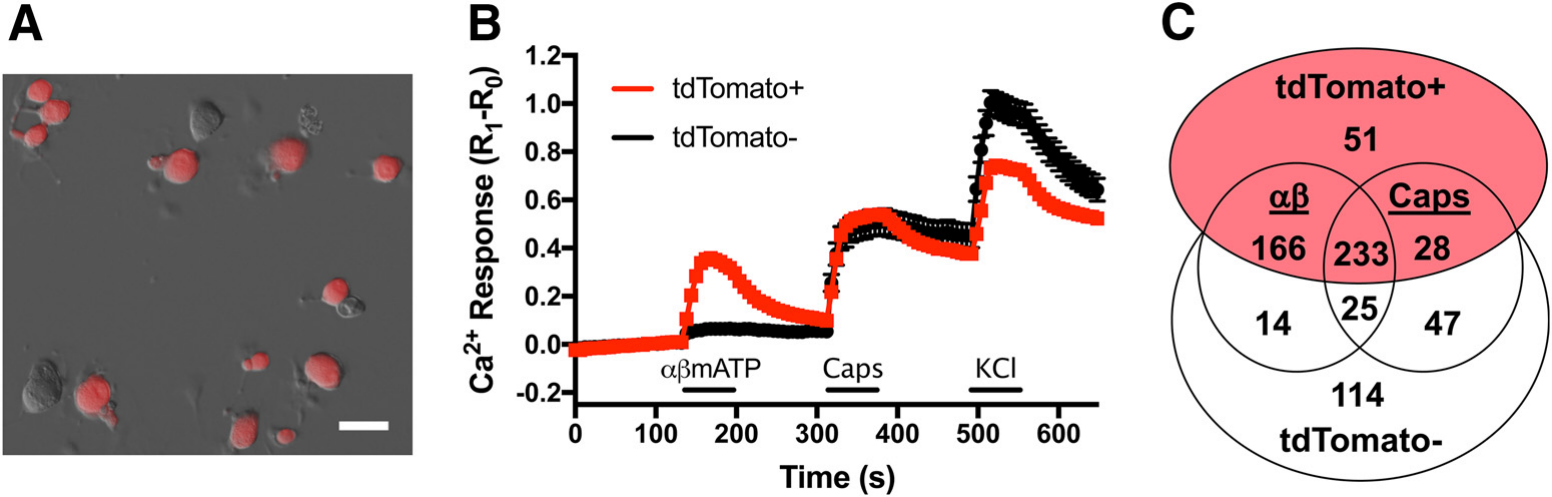
Responsivity to αβmATP correlates with reporter expression in vagal neurons from P2X_2_-tdTomato mice. ***A***, Brightfield image of dissociated vagal neurons overlaid with tdTomato (red) expression. Scale bar: 40 μm. ***B***, Mean ± SEM [Ca^2+^]_i_ responses of tdTomato+ (red, *n* = 478) and tdTomato– (black, *n* = 200) vagal neurons to αβmATP (10 μm), capsaicin (Caps, 1 μm), and KCl (75 mm). ***C***, Euler diagram denoting the number of vagal neurons in each specific subset as determined by tdTomato expression and responsivity to αβmATP and capsaicin.

To link tdTomato expression to P2X_2_ expression in the P2X_2_-tdTomato mouse, we investigated the sensitivity of vagal sensory neurons to the P2X_2/3_ agonist αβmATP. Previous studies indicate that virtually all nodose and jugular neurons express P2X_3_, but P2X_2_ is only expressed in nodose neurons (Cockayne et al., 2005; Kwong et al., 2008; Nassenstein et al., 2010; Surdenikova et al., 2012; Trancikova et al., 2018). αβmATP only causes significant vagal neuron activation in neurons that express both P2X_2_ and P2X_3_ (Lewis et al., 1995; Dunn et al., 2001; Khakh et al., 2001). Here, we assessed Ca^2+^ fluxes in dissociated P2X_2_-tdTomato vagal neurons in response to αβmATP (10 μm) and the TRPV1 agonist capsaicin (1 μm) in 478 tdTomato+ and 200 tdTomato– neurons (Fig. 4). The mean response to αβmATP was significantly greater in tdTomato+ neurons compared with tdTomato– neurons (0.29 ± 0.01 vs 0.06 ± 0.01, *p* < 0.05). Out of the 478 tdTomato+ neurons, 399 neurons responded to αβmATP (83.4%; Fig. 4*C*). Only 39 of the 200 tdTomato– neurons (19.5%) responded to αβmATP (Fig. 4*C*). The mean αβmATP response of αβmATP-sensitive neurons was greater in tdTomato+ neurons than tdTomato– neurons (0.35 ± 0.01 vs 0.22 ± 0.04, *p* < 0.05). There was no difference in mean capsaicin response between tdTomato+ and tdTomato– neurons (0.35 ± 0.02 vs 0.39 ± 0.04, *p* > 0.05). However, there were more capsaicin-sensitive neurons in the tdTomato+ population (261 out of 478, 54.6%) than in the tdTomato– population (72 out of 200, 36.0%). As such, the mean capsaicin response in capsaicin-sensitive neurons was significantly smaller in tdTomato+ neurons compared with tdTomato– neurons (0.66 ± 0.03 vs 1.08 ± 0.09, *p* < 0.05). Overall, the data indicate that tdTomato expression in vagal neurons of P2X_2_-tdTomato mice is a selective marker of αβmATP sensitivity.

**Figure 5.**
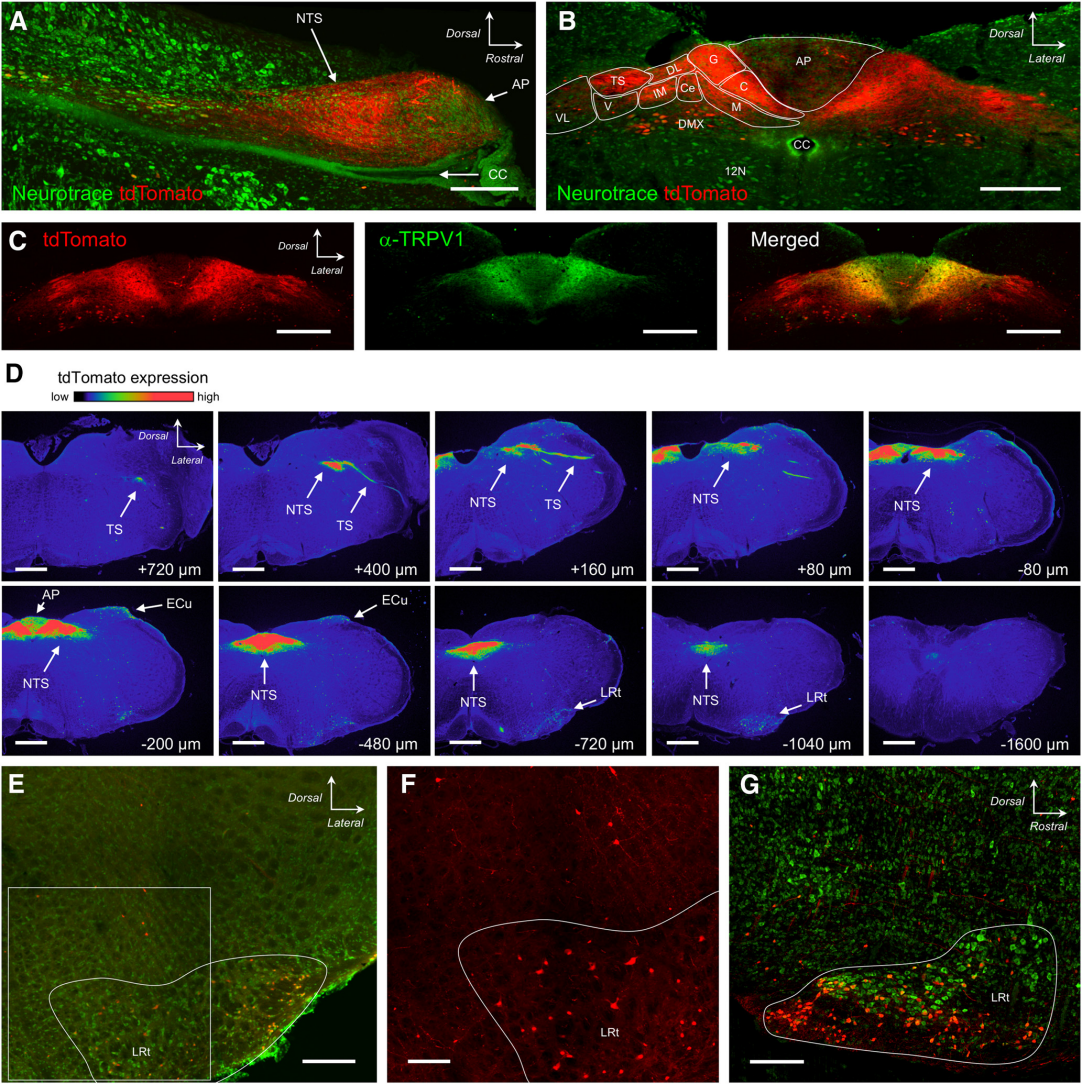
tdTomato expression in the medulla of P2X_2_-tdTomato mice. ***A***, ***B***, tdTomato (red) expression in the dorsal medulla counterstained with NeuroTrace (green). ***A***, Sagittal section at midline. ***B***, Coronal section at −300 μm (relative to obex). ***C***, tdTomato (red) expression counterstained for TRPV1 (green) expression in coronal section at −390 μm (relative to obex). ***D***, tdTomato expression in serial coronal sections from rostral to caudal, with labeling for the position relative to obex. The intensity of native tdTomato expression is shown in rainbow pseudocolor. ***E–G***, tdTomato (red) expression in the ventrolateral medulla counterstained with NeuroTrace (green). ***E***, Coronal section at −540 μm (relative to obex). ***F***, higher magnification of inset in ***E***. ***G***, Sagittal section at 1.08 mm lateral to midline. The following structures are identified: area postrema (AP), central canal (CC), external cuneate nucleus (ECu), dorsal motor nucleus of the vagus (DMX), hypoglossal motor nucleus (12N), lateral reticular nucleus (LRt), nucleus tractus solitarius (nTS), SolC (C), SolCe (Ce), SolDL (DL), SolG (G), SolIM (IM), SolM (M), SolV (V), SolVL (VL), and tractus solitarius (TS). Scale bars: 50 μm (***A***), 300 μm (***B***, ***C***), and 500 μm (***D***).

**Figure 6.**
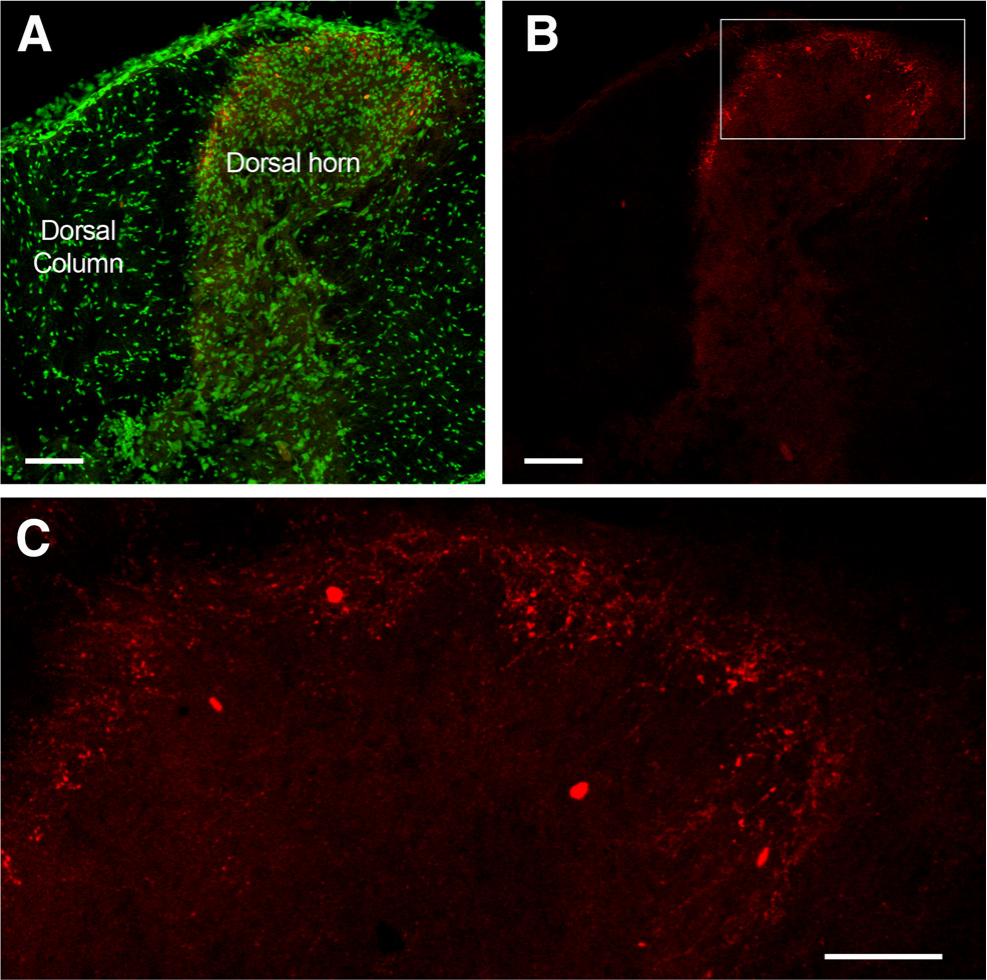
tdTomato expression in the thoracic spinal cord of P2X_2_-tdTomato mice. ***A***, ***B***, Coronal sections with tdTomato expression (red), with (***A***) and without (***B***) counterstain by NeuroTrace (green). ***C***, Higher magnification of superficial dorsal horn laminae. Scale bars:100 μm (***A***, ***B***) and 50 μm (***C***).

Sensory neurons in the vagal, petrosal and geniculate ganglia project central terminals via the tractus solitarius into the nTS in the brainstem medulla. In sections of the medulla of P2X_2_-tdTomato mice, we found robust tdTomato expression in fibers within the tractus solitarius and the nTS, in particular in medial and dorsal subnuclei such as the commissural subnucleus (SolC), gelatinous subnucleus (SolG), dorsal lateral subnucleus (SolDL), medial subnucleus (SolM), intermediate subnucleus (SolIM), and central subnucleus (SolCe; Fig. 5*A*,*B*). We also found some tdTomato+ fibers innervating the ventral subnucleus (SolV) and ventrolateral subnucleus (SolVL) and in the area postrema (Fig. 5*B*). These data are consistent with previous reports that nodose afferents innervate these medulla subnuclei (Kim et al., 2020). Previous studies have also shown that the central projections of TRPV1+ vagal afferents terminate mainly in the medial and dorsal nTS subnuclei and the area postrema (Kim et al., 2020). Here, we found substantial overlap of TRPV1 immunoreactivity and tdTomato within these areas, but the tdTomato+ fibers in lateral and ventral subnuclei did not express TRPV1, thus indicating that P2X_2_+TRPV1+ and P2X_2_+TRPV1– fibers have distinct central terminations (Fig. 5*C*), consistent with electrophysiological recordings of C- and A-fibers (Kubin et al., 1991, 2006). Serial sections indicated that tdTomato+ fibers were found along the entire rostral-caudal axis of the nTS (Fig. 5*D*). There was little tdTomato expression in the medulla other than within the tractus solitarius/nTS/area postrema. In particular, there were only a few sparse tdTomato+ terminations within the spinal trigeminal nucleus and the paratrigeminal complex, consistent with rare tdTomato expression in trigeminal afferent neurons. We did note tdTomato expression in minor subsets of neurons within the dorsal motor nucleus of the vagus and the external cuneate (Fig. 5*B*,*D*). Lastly, we found tdTomato+ neurons within the lateral reticular nucleus (Fig. 5*D-G*), which project mossy fibers to the cerebellum. Only a few tdTomato+ neurons were found in the neighboring caudal ventrolateral medulla (Fig. 5*D-G*). Almost none of the neurons in the rostral ventrolateral medulla expressed tdTomato (Fig. 5*D*). In the thoracic spinal cord, we observed some tdTomato+ fibers within the superficial laminae of the dorsal horn (Fig. 6), consistent with expression of tdTomato in a minor subset of DRG afferents. There was little tdTomato expression in other spinal areas.

**Figure 7.**
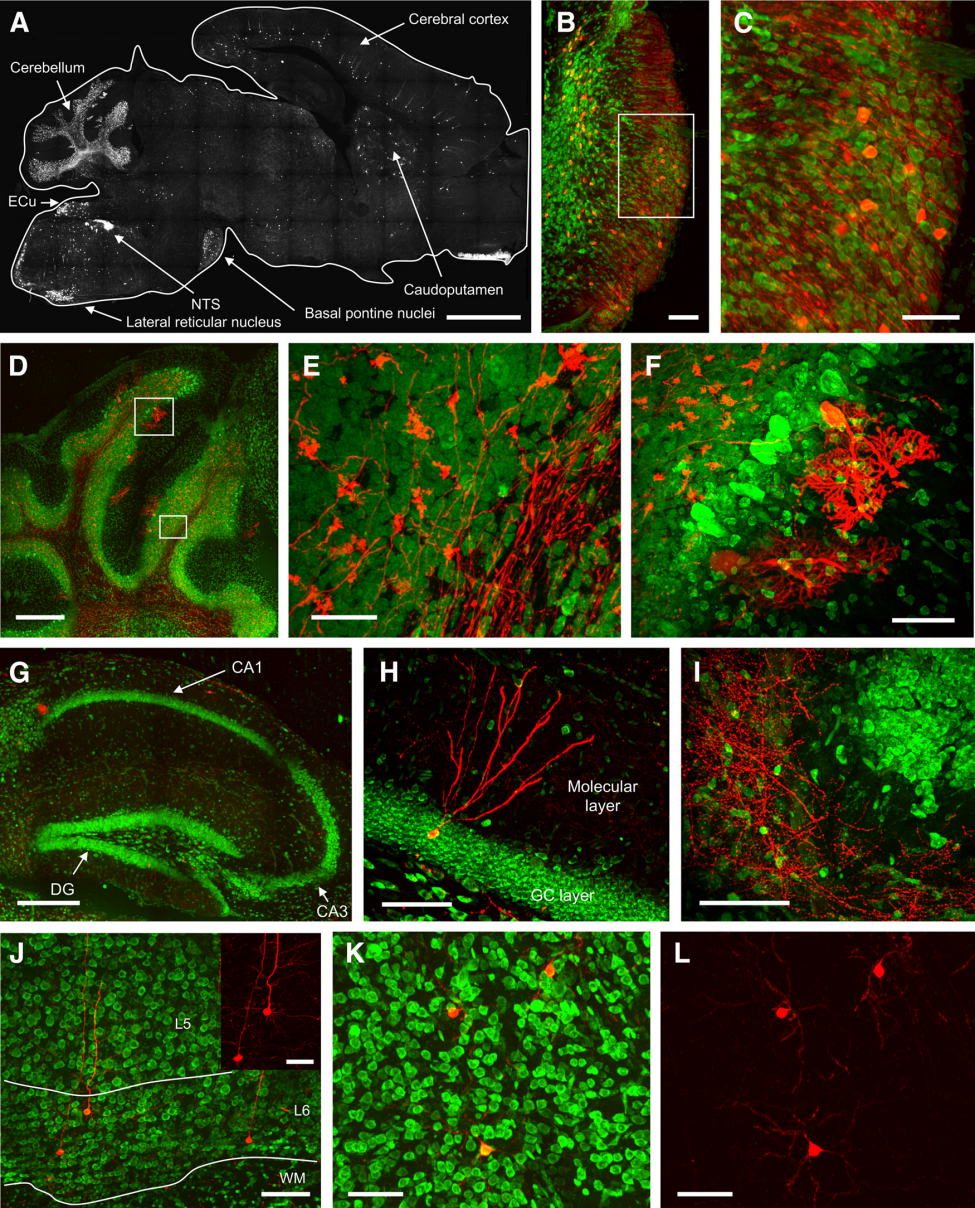
tdTomato expression within the brainstem and brain of P2X_2_-tdTomato mice. ***A***, tdTomato (white) expression in sagittal section at 1 mm lateral to the midline. Ecu, external cuneate nucleus; nTS, nucleus tractus solitarius. ***B–L***, tdTomato (red) expression counterstained with NeuroTrace (green). ***B***, Basal pontine nucleus. ***C***, High magnification of area identified in ***B***. ***D***, Cerebellum. ***E***, High magnification of area identified in ***D*** showing white matter and internal granule layer. ***F***, High magnification of area identified in ***D*** showing internal granule layer, Purkinje cells and the molecular layer. ***G***, Hippocampus. DG, dentate gyrus. ***H***, Suprapyramidal blade of the dentate gyrus. GC layer, granular cell layer. ***I***, Apex of the dentate gyrus. ***J***, Cerebral cortex; inset shows tdTomato expression without NeuroTrace. L5, layer 5; L6, layer 6; WM, white matter. ***K***, ***L***, Caudoputamen with (***H***) and without (***I***) NeuroTrace. Scale bars: 2 mm (***A***), 400 μm (***D***), 300 μm (***G***), 100 μm (***B***, ***J***), 80 μm (***H***, ***I***), 60 μm (***C***, ***F***, ***K***, ***L***), 50 μm (***J***, inset), and 40 μm (***E***).

We extended our investigation of tdTomato expression to the entire brain of P2X_2_-tdTomato mice (Fig. 7). As expected, we again found tdTomato expression in the nTS and the lateral reticular nucleus. Interestingly, we also found tdTomato expression in a subset of neurons within the basal pontine nuclei (Fig. 7*B*,*C*), which also project mossy fibers to the cerebellum. Consistent with this, tdTomato+ mossy fiber axons within the white matter layer and mossy fiber terminations within the molecular layer were observed throughout each lobule of the cerebellum (Fig. 7*D*,*E*). In addition, a minor subset of cerebellar Purkinje cells was tdTomato+ (Fig. 7*F*). No tdTomato+ neurons were observed in the deep cerebellar nuclei. We found very few neurons within the hippocampus expressed tdTomato (Fig. 7*G*,*H*), but some tdTomato+ fibers were noted within the molecular layer surrounding the dentate gyrus granule cell layer (Fig. 7*I*). In the cerebral cortex, tdTomato expression was found in a small number of pyramidal neurons, many of which were found in Layers V and VI. In addition, a small number of neurons in the caudoputamen expressed tdTomato.

**Figure 8.**
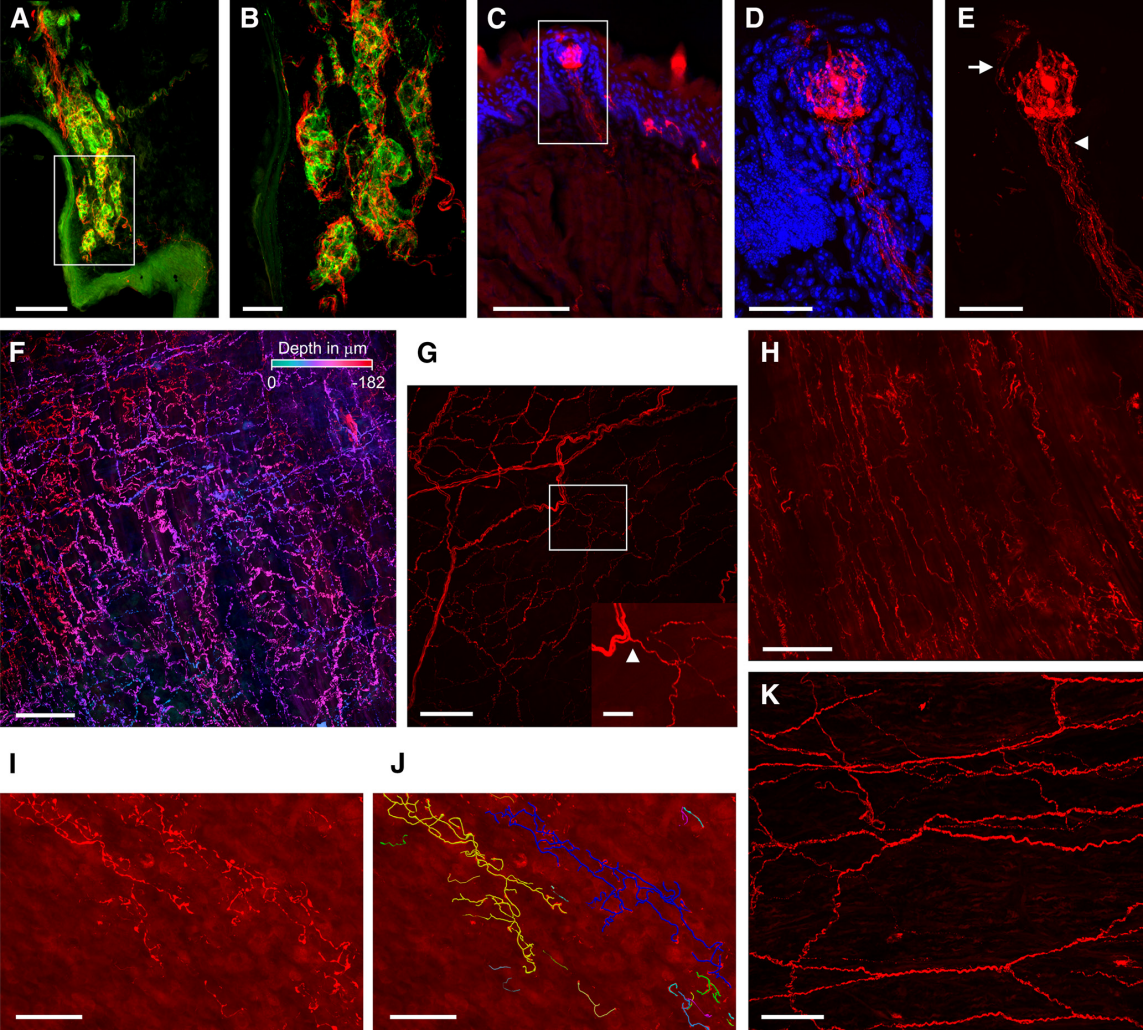
tdTomato expression in nerves innervating carotid bodies, taste buds, trachea, and esophagus of P2X_2_-tdTomato mice. ***A***, Carotid body type 1 cells expressing TH (green) are innervated by nerves expressing tdTomato (red). ***B***, Higher magnification of area identified in ***A***. ***C–E***, Taste bud on the surface of the tongue is innervated by nerves expressing tdTomato (red). DAPI staining (blue) identifies cell nuclei. ***D***, ***E***, Higher magnification of area identified in ***C***. Arrowhead denotes intragemmal fibers, arrow denotes perigemmal fiber. Note the expression of tdTomato in a subset of taste cells within the taste bud. ***F–J***, tdTomato-expressing fibers throughout the epithelial, submucosal, trachealis muscle, and adventitial layers in a wholemount preparation of the trachea. ***F***, Complete *z* projection, with tdTomato expression pseudocolor indicating the *z* depth (total of 182 μm). ***G***, Extensive cabling of tdTomato-expressing fibers (red) in the adventitial layer with some of the muscle layer (63 μm in depth). Inset, Higher magnification of area identified in ***G***. Arrowhead highlights an example of a punctate multibranched fiber branching off a thick bundle of broad axons. ***H***, tdTomato-expressing fibers (red) innervating the trachealis muscle layer (25 μm in depth). ***I***, ***J***, tdTomato-expressing intraepithelial terminations (red) within the epithelial layer. Terminal arborizations were traced using Neurolucida software (***J***). ***K***, tdTomato-expressing fibers (red) in the submucosa and mucosal muscle layers in a wholemount preparation of the esophagus. Scale bars: 100 μm (***A***, ***C***, ***F***, ***G***, ***H***, ***I***, ***J***, ***K***) and 30 μm (***B***, ***D***, ***E***, ***G***, inset).

Next, we investigated tdTomato expression in the carotid body, tongue, trachea, and esophagus, tissues that are thought to be innervated by P2X_2_-expressing afferents (Rong et al., 2003; Finger et al., 2005; Yu et al., 2005; Kwong et al., 2008; Mazzone and Undem, 2016). In the carotid body, we observed dense terminations of glossopharyngeal tdTomato-expressing fibers innervating TH-expressing glomus type 1 cells (Fig. 8*A*,*B*). We found tdTomato-expressing fibers innervating fungiform, filiform, and circumvallate papillae on the tongue (Fig. 8*C–E*). Intragemmal tdTomato+ terminations were observed in fungiform taste buds, along with the occasional perigemmal tdTomato+ terminations. In addition, tdTomato was observed in a subset of cells within the taste buds, which are likely taste cells (Huang et al., 2011). In wholemount preparations of the trachea, we found tdTomato-expressing fibers throughout the entire trachea, although there were far more innervating the dorsal membranous part of the trachea (trachealis muscle regions) than in the anterolateral membranous parts over the cartilaginous rings and ligaments (data not shown). In the membranous portion, tdTomato-expressing fibers were observed from the epithelial layer through to the adventitia (Fig. 8*F*). Distinct patterns of tdTomato-expressing fibers were noted in each layer. In the adventitia, dense cabling of fibers coursed parallel to the epithelial surface (Fig. 8*G*). In places, individual thin, punctate-like fibers were observed branching off the larger tdTomato-expressing axons that appeared to travel in bundles (Fig. 8*G*, inset). Multiple fibers climbed up to the smooth muscle layer, which was densely innervated with a host of parallel fibers following the transversal axis of the trachealis muscle (Fig. 8*H*). We found numerous tdTomato-expressing intraepithelial terminations within the membranous part of the trachea. These tended to have similar structures: parental axons proceeding up through the submucosal layer and then undergoing numerous branching in the subepithelial layer resulting in dense highly arborized structures intercalated with the tracheal epithelium (Fig. 8*I*,*J*). Nerve tracing software was able to show that each branch in the arbor was connected to the same parental axon, but we were unable to determine whether the nearby arbors were derived from the same nerve. Lastly, we observed tdTomato-expressing fibers within the mucosal and submucosal layers of the esophagus (Fig. 8*K*). Often axons ran together through these layers in bundles of more than five axons, but individual punctate-like terminations could also be observed.

**Figure 9.**
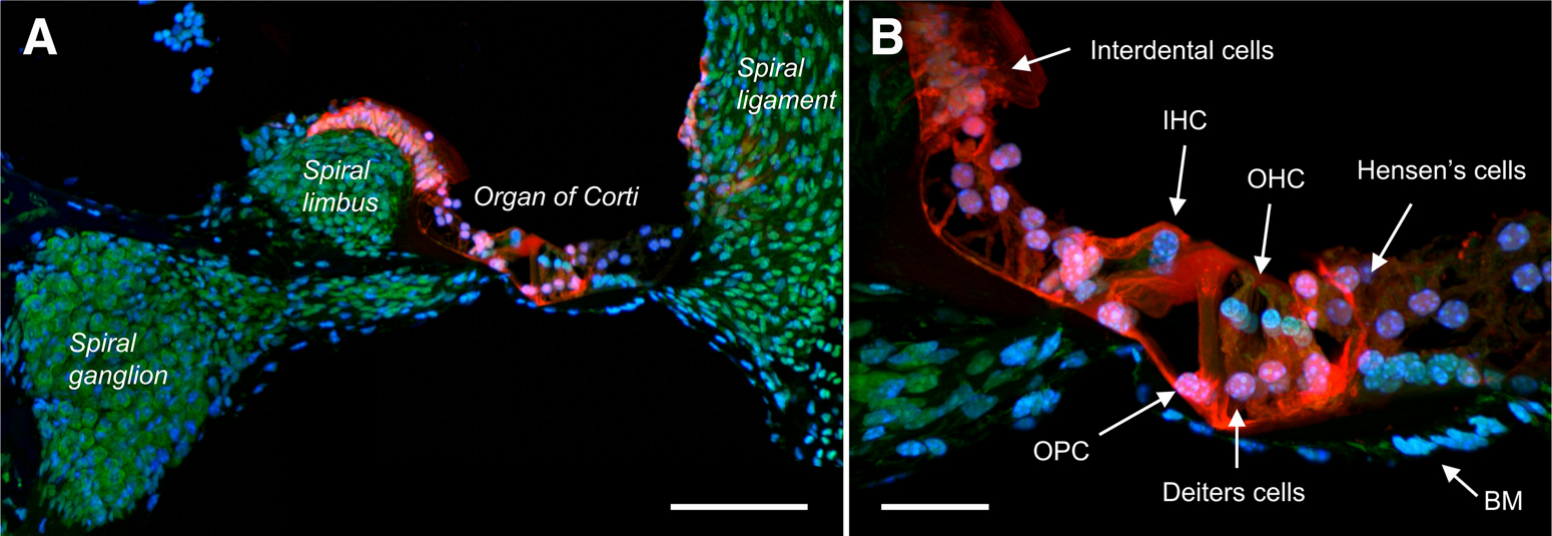
tdTomato expression within the cochlear of P2X_2_-tdTomato mice. ***A***, Section with tdTomato expression (red) including afferent cell bodies in the spiral ganglion, the spiral limbus, spiral ligament, and the organ of Corti, counterstained with NeuroTrace (green) and DAPI (blue). The tectorial membrane is missing from this section. ***B***, Higher magnification of the organ of Corti from ***A***. The following additional structures are identified: basilar membrane (BM), inner hair cells (IHC), outer hair cells (OHC), outer pillar cells (OPC). Scale bars: 100 μm (***A***) and 30 μm (***B***).

Lastly, we investigated the expression of tdTomato in the cochlea, based on reports that P2X_2_ is important for purinergic-mediated adaptation and protection against noise-induced ototoxicity (Yan et al., 2013; Cederholm et al., 2019). We found robust expression of tdTomato in numerous specialized cells within the organ of Corti, including in the inner hair cells, outer hair cells, Deiters cells, Hensen’s cells, and the outer pillar cells (Fig. 9). No tdTomato expression was noted in the basilar membrane. We also found tdTomato expression in the superficial interdental cells on the spiral limbus, and in some cells within the spiral ligament. Importantly, tdTomato was not found in any spiral sensory neurons within the spiral ganglia, nor in their fibers that innervate the hair cells.

## Discussion

P2X_2_ expression has previously been assessed in multiple cell types using immunohistochemistry, *in situ* hybridization, RT-PCR, and functional sensitivity to ATP and other P2X ligands. Although it is generally agreed that P2X_2_ is expressed on at least some peripheral afferent neurons, there is substantial disagreement regarding the precise details of its expression. Here, we have used a genetic approach, producing a knock-in P2X_2_^Cre^ mouse that allows for the visualization of P2X_2_ gene expression systematically. We replaced the endogenous P2X_2_ stop codon with a 2A-Cre cassette. 2A is a self-cleaving peptide, thus expression of Cre recombinase peptides is expected to match P2X_2_ peptide expression on a one-to-one basis. This gene-targeted Cre expression system is more efficient than internal ribosome entry site (IRES) sequences (Furler et al., 2001; Gao et al., 2012) that have often been used in other knock-in Cre reporters.

The vagal ganglia are composed of nodose afferent neurons and jugular afferent neurons, both of which project sensory nerve terminals to multiple visceral organs. We found that almost all nodose neurons expressed tdTomato compared with almost none of the jugular neurons, thus indicating the selective expression of P2X_2_ in the nodose portion of the vagal ganglia. Unlike in guinea pigs and larger mammals, the nodose and jugular ganglion are fused in mice. In these studies, we have, based on the idiosyncratic gross anatomy of each ganglion, subjectively assigned a hard line separating the nodose and jugular portions. This is likely an oversimplification of the distribution of nodose and jugular neurons, which are sometimes not perfectly delineated (Nassenstein et al., 2010; Surdenikova et al., 2012). As such, a small number of P2X_2_+ and P2X_2_– neurons may have been inappropriately assigned. Overall, our data are consistent with single neuron RT-PCR and RNA-seq analysis of nodose and jugular neurons, which show that while P2X_3_ is expressed by almost all vagal neurons, P2X_2_ expression is restricted to nodose neurons (Kwong et al., 2008; Nassenstein et al., 2010; Surdenikova et al., 2012; Wang et al., 2017; Trancikova et al., 2018; Kupari et al., 2019). ATP or αβmATP produces little to no activation of jugular afferents because of the rapid desensitization of homomeric P2X_3_ channels, whereas these purinergic agonists evoke robust activation of dissociated nodose neurons and nodose peripheral terminals because of their persistent activation of heteromeric P2X_2/3_ receptors (Undem et al., 2004; Yu et al., 2005; Kwong et al., 2008; Nassenstein et al., 2010). Indeed, knock-out of P2X_2_ converts ATP-evoked currents in nodose neurons into rapidly desensitizing P2X_3_-like currents (Cockayne et al., 2005). We found that Ca^2+^ influx responses to αβmATP in individual vagal neurons were correlated with tdTomato expression, confirming that the functional presence of P2X_2_-like responses was consistent with P2X_2_ reporter expression. P2X_2_ expression was noted in both TRPV1+ and TRPV1– nodose populations, consistent with previous studies (Undem et al., 2004; Kwong et al., 2008; Taylor-Clark et al., 2015).

Compared with the vagal ganglia, there is greater uncertainty regarding the expression of P2X_2_ in the DRG and trigeminal ganglia. P2X_2_ immunoreactivity was reported to be in the majority of DRG neurons (Petruska et al., 2000b; Cockayne et al., 2005), and this immunoreactivity was absent in P2X_2_ knock-out mice (Cockayne et al., 2005). Nevertheless, using mixed or persistent ATP-evoked currents as a marker of P2X_2_ expression, there are reports that P2X_2_ expression ranges from ∼10% to 50% of DRG neurons (Cockayne et al., 2000, 2005; Dunn et al., 2000; Petruska et al., 2000a,b). Our data indicate that ∼12% of DRG neurons express P2X_2_. This restricted expression is consistent with single neuron RT-PCR and RNA-seq analysis of DRG neurons (Kwong et al., 2008; Surdenikova et al., 2012; Usoskin et al., 2015; Trancikova et al., 2018; Hockley et al., 2019). P2X_2_ immunoreactivity has also been reported to be in a large number of trigeminal neurons (Spehr et al., 2004; Ambalavanar et al., 2005; Simonetti et al., 2006; Staikopoulos et al., 2007). Mixed or persistent ATP-evoked currents have been noted in ∼15–75% of trigeminal neurons (Spehr et al., 2004; Damann et al., 2006; Luo et al., 2006; Simonetti et al., 2006; Liu et al., 2015). Our data indicate that ∼4% trigeminal neurons express P2X_2_. It is possible that P2X_2_’s contribution to “persistent” ATP-evoked currents in trigeminal neurons has been overestimated because of context-dependent P2X_3_ desensitization kinetics (Bianchi et al., 1999; Sokolova et al., 2006) and the contribution of other purinergic channels such as P2X1, P2X4, and P2X5, which are also expressed in trigeminal neurons (Kuroda et al., 2012; Liu et al., 2015).

Previous immunohistochemical and *in situ* hybridization studies have demonstrated widespread and robust P2X_2_ expression throughout the rodent CNS, including in the nTS, area postrema, ventrolateral medulla, medial vestibular nucleus, spinal trigeminal nucleus, hippocampus, hypothalamus, thalamus, striatum, substantia nigra, cerebellum, and cerebral cortex (Kidd et al., 1995; Collo et al., 1996; Vulchanova et al., 1996; Kanjhan et al., 1999; Yao et al., 2000, 2003; Gourine et al., 2003). However, based on the inconsistencies of previous P2X_2_ detection in sensory ganglia, we briefly investigated the CNS expression of P2X_2_ with our reporter mouse. In general, we found P2X_2_ expression in the CNS was limited compared with previous reports. Consistent with the widespread expression of tdTomato in nodose and petrosal neurons we found tdTomato-expressing central terminals within multiple subnuclei throughout the nTS and in the area postrema. Thus, P2X_2_-expressing terminals target the same areas as terminals that express 5HT3 (another nodose neuronal marker that is expressed in very few jugular neurons; Kim et al., 2020). The nTS is the major target of vagal, geniculate, and petrosal ganglia afferents and is involved in reflex control of the cardiovascular, respiratory, and digestive systems. Consistent with the expression of tdTomato in few trigeminal neurons, there was little tdTomato expression in the spinal trigeminal nucleus or the paratrigeminal complex in the medulla. This distinguishes the P2X_2_-tdTomato expression observed here from 5HT3-tdTomato expression, as 5HT3 is expressed in a large number of trigeminal afferents (Kim et al., 2020). This suggests that vagal terminations in the paratrigeminal complex (Driessen et al., 2015; McGovern et al., 2015; Kim et al., 2020), which may play a role in defensive reflexes from the airways, are exclusively via jugular afferents. There was limited tdTomato expression in the thoracic spinal cord, with the majority observed in fibers terminating in the superficial laminae of the dorsal horn. These are most likely to be the central projections of the limited number of DRG afferents that express P2X_2_.

There was little tdTomato expression throughout the rest of the brainstem, with the exception of a subset of neurons in the pontine nucleus and lateral reticular nucleus, both of which project glutamatergic mossy fibers to the cerebellum (Kratochwil et al., 2017). In particular, we found only sparse P2X_2_ expression in the intermediate/caudal ventrolateral medulla and virtually no P2X_2_ expression in the rostral ventrolateral medulla. Previous studies of ATP-evoked modulation of respiratory neurons within these regions have suggested that these actions correlated with P2X_2_ immunoreactivity (Gourine et al., 2003; Yao et al., 2003). P2X_2_ expression in mossy fibers has been reported previously (Collo et al., 1996; Kanjhan et al., 1999), and these channels are thought to be distributed along the projections to the cerebellum (Florenzano et al., 2002). Indeed, we observed robust tdTomato expression in a large number of mossy fibers within the cerebellum. tdTomato expression was limited in other brain regions to very few neurons. Of note, we found few P2X_2_-expressing neurons within the hippocampus, despite immunohistochemical and *in situ* hybridization studies identifying widespread P2X_2_ expression in neurons within the CA1-3 and dentate gyrus (Kidd et al., 1995; Kanjhan et al., 1999), and a transgenic mouse model with a P2X_2_ fusion protein with yellow fluorescent protein identifying P2X expression in hippocampal mossy fibers (Haustein et al., 2014).

We exploited the robust expression of tdTomato throughout P2X_2_-expressing peripheral nerves to study their anatomy. As expected, glossopharyngeal afferents (projected from petrosal neurons) innervating the glomus type 1 cells in the carotid body expressed P2X_2_. Type 1 cells are the principle peripheral chemoreceptors, and P2X_2_ receptors are required for hypoxic signaling (although not CO_2_ detection) from the carotid body (Rong et al., 2003). The structural relationship between the P2X_2_-expressing fibers and the type 1 cells is consistent with previous immunohistochemical studies (Yokoyama et al., 2019). Gustatory afferents innervating taste buds in the tongue expressed P2X_2_, consistent with the critical role of this receptor in taste cell-afferent signaling (Finger et al., 2005). Previous research has also shown P2X_2_ expression in taste buds using RT-PCR, and signaling of taste cell P2X_2_ may act as autocrine, positive feedback signal to amplify taste-evoked ATP secretion (Huang et al., 2011). Our data suggest that P2X_2_ expression occurs in only a subset of taste cells.

We investigated P2X_2_-expressing terminals in the trachea and esophagus, which are likely projected from nodose neurons (Ricco et al., 1996; Yu et al., 2005; Kwong et al., 2008; Prescott et al., 2020). Previous studies of vagal afferent terminations in the mouse trachea using GFP expression following intraganglionic injections of an AAV-GFP construct have identified two classes of vagal afferents in the membranous area of the trachea: dorsal terminal structures that had fibers running largely in parallel to the smooth muscle but did not penetrate the epithelial layer, and small intraepithelial terminals (Hennel et al., 2018). Based on the similar morphology, it is likely that the P2X_2_-expressing terminals observed in the trachea in the current study are a combination of dorsal terminal structures and small intraepithelial terminals, thus suggesting that these terminals are projected by nodose (P2X_2_-expressing) neurons. Interestingly, the major vagal nerve terminal type found in the ligamentous part of the mouse trachea (termed “anterolateral segmental array”) by Hennel et al. (2018) was not labeled in P2X_2_-tdTomato mice, indicating that these probably originate from jugular neurons. Previous tracheal studies have identified A-fiber terminals with complex dendritic arbors innervating the epithelium and subepithelial layers over the cartilage rings in rats (Yamamoto and Nakamuta, 2018) and in the subepithelial layer over the ends of the cartilage rings in guinea pigs (Mazzone et al., 2009). Despite the fact that tracheal A-fibers are likely projected from nodose neurons (Mazzone et al., 2009), they were not detected in either the present study in P2X_2_-tdTomato mice or in the AAV-GFP mouse study by Hennel et al. (2018), suggesting species differences in A-fiber innervation of the trachea. This interpretation is somewhat complicated by the lack of αβmATP sensitivity in nodose Aδ fibers innervating the guinea pig trachea (Canning et al., 2004), suggesting that these nodose fibers surprisingly do not express P2X_2_. However, there are no published reports of P2X_2_ expression in guinea pig nodose neurons labeled from the trachea, so definitive conclusions are not possible at this time. We also observed tdTomato-expressing fibers in the esophageal mucosa, which is innervated by nodose, jugular and DRG afferents (Yu et al., 2005; Surdenikova et al., 2012; Ru et al., 2015). Similar nerve structures were identified in the mouse esophageal mucosa following vagal intraganglionic injections of AAV-GFP (Harsanyiova et al., 2020). Thus, it is likely that the esophageal fibers identified here are projected by nodose (P2X_2_-expressing) neurons.

Lastly, we found evidence of P2X_2_ expression in hair cells and their support cells within the organ of Corti in the cochlea. Previous studies have shown that both inner and outer hair cells express P2X_2_, which mediate ATP-induced cationic currents (Housley et al., 1999; Järlebark et al., 2002). We found no expression of P2X_2_ in the spiral ganglionic afferents that innervate the inner and outer hair cells, in disagreement with some immunohistochemical studies and dissociated patch clamp studies (Järlebark et al., 2002; Weisz et al., 2009; Ito and Dulon, 2010). Knock-out of P2X_2_ has no effect on auditory thresholds in non-aged mice (Yan et al., 2013), indicating that this channel is not critical to neurotransmission between the inner hair cells and the type 1 spiral afferents. Nevertheless, P2X_2_ knock-out accelerates noise-induced ototoxicity (Yan et al., 2013). Noise evokes local ATP release which causes P2X_2_-mediated dampening of the cochlea amplifier by modifying the electromotility properties of the outer hair cells (Yu and Zhao, 2008; Housley et al., 2013; Cederholm et al., 2019). This paracrine humeral purinergic signaling is thought to complement the medial olivocochlear reflex suppression of the cochlear amplifier that is mediated by the type 2 spiral afferents that innervate the outer hair cells (Froud et al., 2015). Our observation of P2X_2_ only in the hair cells and support cells is consistent with recent research that directly implicated the outer hair cell rather than the spiral afferents in the P2X_2_-mediated pathways (Cederholm et al., 2019).

In summary, the P2X_2_-cre reporter mouse demonstrates that P2X_2_ expression is limited to subsets of neurons and specialized cells in the cochlea. Previous reports using biochemical techniques have overestimated the number of cell types that express P2X_2_. This mouse provides a genetic approach to the identification and manipulation of P2X_2_-expressing cell types. The current study used ROSA26-loxP-STOP-loxP-tdTomato mice to visualize cre-mediated recombination in the P2X_2_-cre-expressing cells. It should be noted that the excision of the loxP-flanked stop region on cre expression is irreversible, thus tdTomato expression cannot discriminate between transient and current P2X_2_ expression in a given cell. It is possible that some of the tdTomato expression described here is because of transient P2X_2_ expression.

## Synthesis

Reviewing Editor: Douglas Bayliss, University of Virginia School of Medicine Decisions are customarily a result of the Reviewing Editor and the peer reviewers coming together and discussing their recommendations until a consensus is reached. When revisions are invited, a fact-based synthesis statement explaining their decision and outlining what is needed to prepare a revision will be listed below.

Synthesis

This manuscript was seen by two reviewers, who note that this work nicely describes the development of a useful new mouse model -- P2X2-Cre mice -- that was used in conjunction with Cre-dependent reporter mice to provide new information on localization of P2X2 expression. As you will see from the reviewer comments (appended below), the suggestions for improvement largely involve addressing issues of data presentation, clarifying text, and providing additional citations to relevant work. A further point raised during consultation involved the need to explicitly acknowledge the limitations of this Cre-reporter approach for localizing sites of gene expression (e.g., the reporter can reveal any site where P2X2-Cre was ever expressed, even if those cells are not currently expressing P2X2). This should also include a brief discussion of how those limitations affect interpretations vis a vis discrepancies with other methods used for P2X2 detection.

Please find the full comments appended below.

Reviewer #1

Advances the Field

Development of a novel mouse for studying P2X2 biology. Clarification of P2X2 expression.

Comments

This paper is a relatively straight forward descriptive study describing the development of a P2X2-Cre transgenic mouse. By crossing this new line with a Cre-dependent reporter line, the authors have characterized P2X2 expression throughout the nervous system. The study clarifies some misinformation in the literature with respect to P2X2 distribution, largely driven by immunohistochemical studies with seemingly non-selective antisera. Of note is the confirmed limited expression in the dorsal root and jugular sensory ganglia, predicted from functional studies. Given the descriptive nature of the study, it is difficult to find major criticism. The new mouse line will be of value to those interested in studying P2X2 function. Some relatively minor comments for the authors to consider:

1.The authors state that the mice have “no apparent phenotype” (line 109). Was this formally assessed or from simple observation?

2.The definition of nodose and jugular portions of the vagal ganglia seem arbitrary. Other than a line drawn across the photomicrograph I can’t see any evidence of what criteria the authors used to distinguish these two populations of cells. No selective markers were used. This is an important consideration when determining which cells express P2X2 in these two tissues. The few P2X2 positive cells in the nominated jugular portion of the ganglia could in fact be misplaced nodose neurons and vice versa for the P2X2 negative cells in the nodose (given the fused nature of these tissues). Have the authors attempted to use more selective markers to define nodose and jugular neurons (e.g. Phox2b, Prdm12 or others?). This might change the interpretation of the data slightly.

3.Line 266 the authors state “The mean response of tdTomato+ neurons to a,b-mATP was significantly greater in tdTomato+ neurons compared to tdTomato- neurons (0.29+/-0.01 vs. 0.06+/-0.01, p<0.05).” Please correct the repetition of wording here (ie delete one mention of TdTomato+). The sentence follows on with a comparison of capsaicin sensitivity. However, it is unclear if this is a comparison of mean responsiveness in all neurons tested or only those that they determined were capsaicin sensitive? (ie not all neurons would be expected to be capsaicin sensitive). Please clarify. Finally, why do the authors suppose that only 83% of TdTomato positive neurons showed a,b-mATP sensitivity? Is this a dose issue?

4.The expression of P2X2 in the nTS is consistent with the expression in the nodose ganglia, although the authors should note that there are other inputs to the nTS in addition to vagal. Without a full investigation they cannot rule out other P2X2-positive sensory or central neural inputs.

5.Line 289 the authors state “and ventral subnuclei did not express TRPV1, thus indicating that P2X2+TRPV1+ and P2X2+TRPV1- fibers have distinct central terminations”. In many ways this is already known from the many studies of nodose inputs to the nTS over many years. Some acknowledgement that the present data is merely consistent with other studies of capsaicin-responsive units in the nTS is needed here.

6.Line 294 states “trigeminal nucleus and the paratrigeminal complex, consistent with rare tdTomato expression in trigeminal afferent neurons”. The authors should note that jugular and DRG neurons terminate in the paratrigeminal/ trigeminal nuclear complex as well. This is also consistent with their data.

7.I was surprised not to see inclusion of spinal cord sections in the distribution characterization, given the DRG data presented. This would be useful. I was also surprised not to see inner ear, given the evidence for P2X2 homomers in tuning the cochlear. This would have been an interesting and validating inclusion. Do the authors have any of these materials for addition?

8.Line 414 states “tdTomato expression observed here from 5HT3-tdTomato expression, as 5HT3 is expressed in a large number of trigeminal afferents (Kim et al., 2020). This suggests that vagal terminations in the paratrigeminal complex (Kim et al., 2020), which may play a role in defensive reflexes from the airways (Driessen et al., 2015), are exclusively via jugular afferents.” This reviewer is pleased to see the authors correct the previous over-interpretation of the cited data. It would be best to acknowledge here also that this is consistent with previous studies in rats and guinea pigs that assessed this question directly, unlike Kim et al or the present study which can only make inferences.

9.Line 455 “Interestingly, the major vagal nerve terminal type found in the ligamentous part of the mouse trachea (termed ‘anterolateral segmental array’) by Hennel et al. (2018) was not labeled in P2X2-tdTomato mice, indicating that these probably originate from jugular neurons. Previous tracheal studies have identified A-fiber terminals with complex dendritic arbors innervating the....Despite the fact that these tracheal A-fibers are likely projected from nodose neurons (Mazzone et al., 2009), they were not detected in either the present study in P2X2-tdTomato mice or in the AAV-GFP mouse study by Hennel et al. (2018), suggesting species differences...” Whilst I don’t disagree with the suggestion of species differences (rats also lack the complex endings labeled by FM2-10 in the Mazzone et al 2009 study), the interpretation may need qualifying as, at least in guinea pigs, the nodose terminals referred to lack functional responsiveness to a,b-mATP suggesting they are an unusual nodose mechanoreceptor subtype. Hence, it could be that some nodose neurons innervating the trachea lack P2X2 expression, thereby presenting with a jugular-like molecular appearance in this regard. The authors did not fully characterize these terminals and thus cannot confidently rule this out.

Reviewer #2

Advances the Field

This study describes the development of a mouse reporter strain for ATP-gated ionotropic P2X2 receptor subunit. The new mouse strain provides information about specific expression of this receptor and is important for our understanding of the physiological role of purinergic signalling mediated by ATP acting at receptors that contain P2X2 subunit. The data reported are solid and convincing and the paper is very well written.

Comments

This study describes the development of a mouse reporter strain for ATP-gated ionotropic P2X2 receptor subunit. The new mouse strain provides information about specific expression of this receptor subunit and is important for our understanding of the physiological role of purinergic signalling mediated by ATP acting at receptors that contain P2X2 subunit. The data reported are solid and convincing (the reported pattern of P2X2 expression is very similar to what was observed by this reviewer in the dorsal brainstem and the carotid body with the use of anti-P2X2 antibody from Alamone) and the paper is very well written. I only have a few minor comments for the authors to consider:

1. It would be of interest to see the expression of P2X2 in the petrosal ganglion where the cell bodies of the carotid sinus afferents are located;

2. Perhaps for completeness the authors may consider citing two additional earlier studies that reported expression of the P2X2 receptor subunit in the central nervous system (PMID: 14521999; PMID: 12878756);

3. P2X2 receptor is one of only a few of known ionotropic receptors that are sensitive to changes in pH within the physiological range (PMID: 8730726). It was suggested some 20 years ago that P2X2 receptor may be important for pH dependent control of breathing at the level of the ventral brainstem and the expression of P2X2 was shown to be present in functionally identified respiratory neurons (PMID: 12878756). Considering the vital importance of this behaviour it would be of interest to see at higher magnification the cluster of neurons expressing P2X2 in the ventral region of the medulla (images 6 and 7 of Figure 5D).

## Author Response

Authors’ response: We thank the reviewers and editor for their critique of our manuscript. We appreciate their positive comments and, having made the requested changes, we hope that the manuscript is now suitable for publication.

This manuscript was seen by two reviewers, who note that this work nicely describes the development of a useful new mouse model -- P2X2-Cre mice -- that was used in conjunction with Cre-dependent reporter mice to provide new information on localization of P2X2 expression. As you will see from the reviewer comments (appended below), the suggestions for improvement largely involve addressing issues of data presentation, clarifying text, and providing additional citations to relevant work. A further point raised during consultation involved the need to explicitly acknowledge the limitations of this Cre-reporter approach for localizing sites of gene expression (e.g., the reporter can reveal any site where P2X2-Cre was ever expressed, even if those cells are not currently expressing P2X2). This should also include a brief discussion of how those limitations affect interpretations vis a vis discrepancies with other methods used for P2X2 detection.

Authors’ response: We agree, and have added text that emphasizes the issue that the floxed-STOP-tdTomato reporter is irreversibly modified by Cre expression, thus cannot discriminate between transient and current P2X2 expression.

Reviewer #1

This paper is a relatively straight forward descriptive study describing the development of a P2X2-Cre transgenic mouse. By crossing this new line with a Cre-dependent reporter line, the authors have characterized P2X2 expression throughout the nervous system. The study clarifies some misinformation in the literature with respect to P2X2 distribution, largely driven by immunohistochemical studies with seemingly non-selective antisera. Of note is the confirmed limited expression in the dorsal root and jugular sensory ganglia, predicted from functional studies. Given the descriptive nature of the study, it is difficult to find major criticism. The new mouse line will be of value to those interested in studying P2X2 function. Some relatively minor comments for the authors to consider:

1.The authors state that the mice have “no apparent phenotype” (line 109). Was this formally assessed or from simple observation?

Authors’ response: This was a simple observation. This has been clarified in the text.

2.The definition of nodose and jugular portions of the vagal ganglia seem arbitrary. Other than a line drawn across the photomicrograph I can’t see any evidence of what criteria the authors used to distinguish these two populations of cells. No selective markers were used. This is an important consideration when determining which cells express P2X2 in these two tissues. The few P2X2 positive cells in the nominated jugular portion of the ganglia could in fact be misplaced nodose neurons and vice versa for the P2X2 negative cells in the nodose (given the fused nature of these tissues). Have the authors attempted to use more selective markers to define nodose and jugular neurons (e.g. Phox2b, Prdm12 or others?). This might change the interpretation of the data slightly.

Authors’ response: We agree that the line is arbitrary, although based upon the idiosyncrasies of the fused ganglion. We have not compared with Phox2b or Prdm12. We have added text to clarify this issue.

3.Line 266 the authors state “The mean response of tdTomato+ neurons to a,b-mATP was significantly greater in tdTomato+ neurons compared to tdTomato- neurons (0.29+/-0.01 vs. 0.06+/-0.01, p<0.05).” Please correct the repetition of wording here (ie delete one mention of TdTomato+). The sentence follows on with a comparison of capsaicin sensitivity. However, it is unclear if this is a comparison of mean responsiveness in all neurons tested or only those that they determined were capsaicin sensitive? (ie not all neurons would be expected to be capsaicin sensitive). Please clarify. Finally, why do the authors suppose that only 83% of TdTomato positive neurons showed a,b-mATP sensitivity? Is this a dose issue?

Authors’ response: The first sentence has been corrected. We thank the reviewer for querying the capsaicin responses. The data provided in the manuscript was the mean capsaicin response of the total tdTomato+ and tdTomato- populations regardless of capsaicin sensitivity. We have added data to show that more tdT+ neurons were capsaicin-sensitive compared to tdT- neurons, and that the capsaicin response of capsaicin-sensitive neurons in the tdT- population was greater than in the tdTomato+ population. We do not have a definitive reason for the fact that only 83% of tdT+ neurons responded in the Ca2+ imaging assay. The definition of a responder is based upon previous studies defining a threshold for the Ca2+ response which is to some degree arbitrary. Populational Ca2+ responses even to ionotropic receptor activation (e.g. ATP on P2X or capsaicin on TRPV1) are a continuum and it can be debatable what is a truly negative response. This can obviously be affected, as the reviewer points out, by dose. 10uM is not a heroic dose (EC50 is reported to be ∼3-9uM) and it is plausible that some P2X2/3-expressing neurons do not pass the threshold. It is obviously important to balance the possibility of false negatives by the possibility of false positives. We would also add that acute dissociation of the neurons may have affected their P2X expression. We have added references to establish the effectiveness of the dose used in this study.

4.The expression of P2X2 in the nTS is consistent with the expression in the nodose ganglia, although the authors should note that there are other inputs to the nTS in addition to vagal. Without a full investigation they cannot rule out other P2X2-positive sensory or central neural inputs.

Authors’ response: We agree. We have amended the text accordingly.

5.Line 289 the authors state “and ventral subnuclei did not express TRPV1, thus indicating that P2X2+TRPV1+ and P2X2+TRPV1- fibers have distinct central terminations”. In many ways this is already known from the many studies of nodose inputs to the nTS over many years. Some acknowledgement that the present data is merely consistent with other studies of capsaicin-responsive units in the nTS is needed here.

Authors’ response: We have amended the text to reference electrophysiological studies that previously addressed the termination of afferent subsets in the nTS.

6.Line 294 states “trigeminal nucleus and the paratrigeminal complex, consistent with rare tdTomato expression in trigeminal afferent neurons”. The authors should note that jugular and DRG neurons terminate in the paratrigeminal/ trigeminal nuclear complex as well. This is also consistent with their data.

Authors’ response: We agree that afferents other than trigeminal terminate in the paratrigeminal complex. We have modified the discussion of the nodose vs. jugular paratrigeminal link accordingly (also see below).

7.I was surprised not to see inclusion of spinal cord sections in the distribution characterization, given the DRG data presented. This would be useful. I was also surprised not to see inner ear, given the evidence for P2X2 homomers in tuning the cochlear. This would have been an interesting and validating inclusion. Do the authors have any of these materials for addition?

Authors’ response: We have added images of the spinal cord, showing that P2X2-expressing fibers terminate in the superficial laminae of the spinal cord. We have added images of the mouse cochlea. As expected, we observed tdTomato expression in hair cells and support cells, but not in spiral afferent neurons. Recent evidence suggests that the role of P2X2 in protecting against noise-induced ototoxicity is due to ATP-evoked modulation of outer hair cell electromotility, rather than a direct activation of afferents.

8.Line 414 states “tdTomato expression observed here from 5HT3-tdTomato expression, as 5HT3 is expressed in a large number of trigeminal afferents (Kim et al., 2020). This suggests that vagal terminations in the paratrigeminal complex (Kim et al., 2020), which may play a role in defensive reflexes from the airways (Driessen et al., 2015), are exclusively via jugular afferents.” This reviewer is pleased to see the authors correct the previous over-interpretation of the cited data. It would be best to acknowledge here also that this is consistent with previous studies in rats and guinea pigs that assessed this question directly, unlike Kim et al or the present study which can only make inferences.

Authors’ response: We have added references related to jugular innervation of the paratrigeminal complex in rats and guinea pigs.

9.Line 455 “Interestingly, the major vagal nerve terminal type found in the ligamentous part of the mouse trachea (termed ‘anterolateral segmental array’) by Hennel et al. (2018) was not labeled in P2X2-tdTomato mice, indicating that these probably originate from jugular neurons. Previous tracheal studies have identified A-fiber terminals with complex dendritic arbors innervating the....Despite the fact that these tracheal A-fibers are likely projected from nodose neurons (Mazzone et al., 2009), they were not detected in either the present study in P2X2-tdTomato mice or in the AAV-GFP mouse study by Hennel et al. (2018), suggesting species differences...” Whilst I don’t disagree with the suggestion of species differences (rats also lack the complex endings labeled by FM2-10 in the Mazzone et al 2009 study), the interpretation may need qualifying as, at least in guinea pigs, the nodose terminals referred to lack functional responsiveness to a,b-mATP suggesting they are an unusual nodose mechanoreceptor subtype. Hence, it could be that some nodose neurons innervating the trachea lack P2X2 expression, thereby presenting with a jugular-like molecular appearance in this regard. The authors did not fully characterize these terminals and thus cannot confidently rule this out.

Authors’ response: We have clarified that guinea pig nodose Adelta fibers innervating the trachea do not respond to P2X ligands and we have added a note of caution to the interpretation.

Reviewer #2

This study describes the development of a mouse reporter strain for ATP-gated ionotropic P2X2 receptor subunit. The new mouse strain provides information about specific expression of this receptor and is important for our understanding of the physiological role of purinergic signalling mediated by ATP acting at receptors that contain P2X2 subunit. The data reported are solid and convincing and the paper is very well written. This study describes the development of a mouse reporter strain for ATP-gated ionotropic P2X2 receptor subunit. The new mouse strain provides information about specific expression of this receptor subunit and is important for our understanding of the physiological role of purinergic signalling mediated by ATP acting at receptors that contain P2X2 subunit. The data reported are solid and convincing (the reported pattern of P2X2 expression is very similar to what was observed by this reviewer in the dorsal brainstem and the carotid body with the use of anti-P2X2 antibody from Alamone) and the paper is very well written. I only have a few minor comments for the authors to consider:

1. It would be of interest to see the expression of P2X2 in the petrosal ganglion where the cell bodies of the carotid sinus afferents are located;

Authors’ response: We have added a representative image of the petrosal ganglion. As expected many of the neurons expressed tdTomato.

2. Perhaps for completeness the authors may consider citing two additional earlier studies that reported expression of the P2X2 receptor subunit in the central nervous system (PMID: 14521999; PMID: 12878756);

Authors’ response: We have added these citations.

3. P2X2 receptor is one of only a few of known ionotropic receptors that are sensitive to changes in pH within the physiological range (PMID: 8730726). It was suggested some 20 years ago that P2X2 receptor may be important for pH dependent control of breathing at the level of the ventral brainstem and the expression of P2X2 was shown to be present in functionally identified respiratory neurons (PMID: 12878756). Considering the vital importance of this behaviour it would be of interest to see at higher magnification the cluster of neurons expressing P2X2 in the ventral region of the medulla (images 6 and 7 of Figure 5D).

Authors’ response: We have added higher magnification images of the ventral lateral region of the medulla. We find only sparse expression of tdTomato in the caudal regions and virtually none in the rostral regions. We have added a point in the discussion relating our findings to the references mentioned by the reviewer.
